# From genome integrity to cancer

**DOI:** 10.1186/s13073-019-0617-y

**Published:** 2019-01-29

**Authors:** Serena Nik-Zainal

**Affiliations:** 1 Department of Medical Genetics, Addenbrooke’s Treatment Centre, The Clinical School, Cambridge Biomedical Campus, Cambridge, CB2 0QQ UK; 2 MRC Cancer Unit, Hutchison/MRC Research Centre, University of Cambridge, Cambridge Biomedical Campus, Cambridge, CB2 0XZ UK

The genome in each of our normal cells acquires somatic genetic changes throughout life [1]. The burden of DNA damage from exogenous and endogenous sources is considerable, but highly competent DNA repair pathways operate in concert to maintain genome integrity [1].

By contrast, the genomes of cancerous cells are a source of biological information regarding cancer development, identifying DNA repair pathways that have gone awry and unveiling excessive DNA damage from external sources [1–3]. They may even serve as a record of physiological processes that are acting inappropriately (e.g., replication stress) [4, 5]. These genomes provide a read-out of the pathological processes that operate in cells as they transition from normality towards malignancy. The read-out can be seen as patterns of mutations, or mutational signatures, at the single nucleotide variant (SNV) level [1–3], as insertions/deletions (indels), as structural variation [4, 5], and even as chromosomal copy number changes [4, 5]. It can manifest as physical abnormalities such as the presence of micronuclei. In other words, outcomes can be appreciated across a whole spectrum of resolutions, ranging from base-pair changes to alterations on the chromosomal scale [5]. The loss of genome integrity is thus highly informative [6], revealing why a tumor has formed and, crucially, how we can potentially exploit these processes in interventions.

This special issue of *Genome Medicine* highlights the advances made in understanding how compromised genome integrity impacts genome architecture, tumor biology, immune escape, and the mechanisms underlying differential response and resistance to cancer therapies, outlining new avenues for precision therapies and clinical decision-making. For example, at base pair resolution, a high frequency of SNVs with characteristic C > A and T > C mutations, together with a high degree of indel formation at polynucleotide repeat tracts (also termed microsatellite instability), has been found in tumors with mismatch repair deficiencies (MMRd) [7]. It is important to detect tumors that have MMRd because of their reported sensitivities to immunotherapy [8, 9], regardless of the cell of origin. Tumors with alternative, distinctive C > A, C > T, and T > G mutations that are associated with activating mutations in the DNA polymerase epsilon gene (*POLE*) have also been suggested to be sensitive to checkpoint inhibitors [10, 11].

These examples of nucleotide-resolution genomic instability contrast with those associated with germline deficiencies in the genes of the homology-directed repair (HR) pathway, such as *BRCA1* and *BRCA2* [2, 4, 12]. In patients with *BRCA1*/*BRCA2* deficiencies, mutational patterns at the SNV and indel levels are reported in conjunction with structural variations and changes at the chromosomal copy number level [4, 12]. Thus, the loss of genome integrity occurs at all levels of genomic resolution in HR-deficient tumors. These pathognomonic patterns of genomic instability have been exploited; for example, algorithms have been developed to identify sporadic tumors with BRCA1/BRCA2 phenotypes in patients who are not germline BRCA1/BRCA2 mutation carriers [12]. Intriguingly, these efforts revealed that the proportion of tumors that have acquired such deficiencies is much larger than was previously appreciated. This is significant because it means that patterns of genomic instability can be used as biomarkers to identify additional tumors that are potentially sensitive to therapeutics originally designed for germline BRCA1/BRCA2-deficient tumors, such as poly (ADP-ribose) polymerase (PARP) inhibitors. Furthermore, these tumors need not be restricted to breast and ovarian cancers and could involve other cancer types too.

The loss of genomic integrity can be influenced by a variety of additional physiological processes, such as transcription and replication [13], the formation of R-loops [14–16], and epigenomic dysregulation [17]. In due course, these additional layers of genomic information will be added to the mix, informing our understanding of the burden and distribution of mutagenesis and of how we can exploit this new knowledge for patient benefit.

A critical feature of cells that have on-going genomic instability is the higher likelihood that new and potentially therapy-resistant clones will be generated. In this special issue, we read how loss of Jun promotes resistance to the histone deacetylase inhibitor entinostat in luminal breast cancer through Myc signaling. Entinostat is in phase III trials for patients with metastatic estrogen-receptor-positive breast cancer, and thus Jun and MYC may represent biomarkers of entinostat responsiveness in breast cancer [18]. Evolvability can arise through the disruption of critical genes or pathways, or through the development of properties that permit cells to escape immune-surveillance [19] and thus promote immune evasion. This area of research has already shown considerable promise, and much work has gone into exploring genomic instability and how it affects evolvability following therapeutic intervention [20, 21].

One area that remains poorly studied is whether there are patterns of genomic instability that distinguish normal cells from cancer cells. Until recently, much of the analysis of mutational patterns has been focused on cancers that have arisen from some common ancestor and thus are clonal in origin. The ability to study the genomic integrity of single cells has been restricted to copy number variation because of the limitations of the relevant technology. In due course, patterns of genomic instability that are perfectly well tolerated in normal cells may be revealed. By contrast, there may be typical forms of loss of genomic integrity that are associated with malignancy or even with poor treatment response and prognosis outcome.

Finally, to tolerate the high burden of mutagenesis, tumors have developed intrinsic properties that permit enduring survival, which are undoubtedly molded by the tumor microenvironment and the patient’s immune response [22]. Human cancers may thus be addicted to features such as checkpoint bypass or may be dependent on alternative components of DNA repair for their survival. These features, however, make cancer cells selectively targetable for therapeutic intervention. The principle of synthetic lethality is predicated on this very point: tumor cells that are null for error-free HR repair are wholly dependent on alternative ways of fixing single-strand breaks. PARP inhibition exploits this point, so that tumor cells are selectively sensitive to PARP-inhibiting drugs and consequently better tolerated in patients. Other synthetic lethality relationships may well exist and remain to be discovered and exploited for the development of new drugs. Using synthetic lethality screening, Bernards and colleagues [23] show that the unfolded protein response pathway may serve as a new potential target for drug-resistant KRAS-mutant colorectal cancers.

The loss of genome integrity, if producing a characteristic pattern, could be used as a marker for prognosis or as a read-out for the stratification of cancer patients. In fact, such patterns could be used even in the absence of an identified genetic or epigenetic driver. In other words, using patterns of loss of genomic integrity could serve as an additional tool in the tool box of diagnostic clinical genomics. We can look forward to a future when a patient’s treatment will be informed by the biological abnormalities that are present in their tumor, based on the general patterns of loss of genomic integrity and not simply on a binary decision of whether a driver mutation is present or not. The profiling of circulating tumor DNA (ctDNA) is becoming increasingly important in clinical oncology, with ctDNA profiling methods being developed and applied in the patient setting for monitoring disease [24] and for capturing the landscape of metastatic disease [25].

When we have gained a deeper understanding of the factors that are required to maintain genomic integrity and have developed greater insights into the causes and consequences of genomic instability, and of course have validated these ideas within clinical trials, perhaps comprehensive genomics will become an imperceptible part of every patient’s diagnostic work-up. Like routine blood screening, a staging computed tomography (CT) scan, or a positron emission tomography (PET) scan, genomics could become an accepted (and even necessary) screening tool that informs patient care.

## Abbreviations

ctDNA: Circulating tumor DNA
HR: Homology-directed repair
indel: Insertion/deletion
MMRd: Mismatch repair deficiency
PARP: Poly (ADP-ribose) polymerase
SNV: Single nucleotide variant
